# Common clinical features of unilateral retinal pigment epithelium dysgenesis and combined hamartoma of the retina and retinal pigment epithelium

**DOI:** 10.1186/s12886-022-02244-x

**Published:** 2022-01-15

**Authors:** Zhe Zhu, Jun Xiao, Lifu Luo, Bo Yang, He Zou, Chenchen Zhang

**Affiliations:** Medical Retina, Eye Center of the Second Hospital of Jilin University, Room 304, 3rd Floor, Out patient Building, No.218, Ziqiang Street, Nanguan District, Changchun, Jilin Province China

**Keywords:** Combined hamartoma of the retina and the retinal pigment epithelium, Unilateral retinal pigment epithelium dysgenesis, Case report

## Abstract

**Background:**

Herein, we report two cases of unilateral retinal pigment epithelium dysgenesis (URPED) in Chinese patients and explore the relationship between URPED and combined hamartoma of the retina and retinal pigment epithelium (CHRRPE).

**Case presentation:**

The lesion margins in the two cases showed pathognomonic clinical features of URPED, namely, a scalloped reticular margin in hyperplastic retinal pigment epithelium and mild fibrosis. The hypoautofluorescence observed by fundus autofluorescence was inverted compared with that observed by fundus fluorescence angiography. A large amount of fibroglial proliferation and disorganization of the retina involving the whole layer, which are also found in peripapillary CHRRPE, were found in the lesions.

**Conclusions:**

URPED appears to share some clinical features with CHRRPE, and the relationship between URPED and CHRRPE needs further study.

**Supplementary Information:**

The online version contains supplementary material available at 10.1186/s12886-022-02244-x.

## Background

Unilateral retinal pigment epithelium dysgenesis (URPED) was first described by Cohen et al. as unilateral, idiopathic leopard-spot lesions in the retinal pigment epithelium (RPE), with fibrosis and hyperplastic changes of the RPE at the margin of the lesion and atrophy of the RPE in the center [1]. The characteristic clinical features of the disease are the distinctive scalloped margin of reticular RPE hyperplasia and fibrosis changes and the remarkable dark reticular pattern presented on fundus autofluorescence (FAF), which is inverted relative to the hyperfluorescence observed with fundus fluorescence angiography (FFA) [2].

The prognosis of this disease is still unclear. When the lesion progresses into the macular region or is accompanied by complications such as choroidal neovascularization (CNV) or epiretinal membrane (ERM), visual impairment can occur [2–6]. In addition, the relationship between URPED and combined hamartoma of the retina and RPE (CHRRPE) remains unclear. Some authors postulated that URPED may be a forme fruste of CHRRPE [3]. However, other scholars believe that URPED and CHRRPE are different diseases given the obvious imaging differences between them [2, 5, 6].

In this study, we hope to further increase understanding of URPED by providing a detailed description of two cases and explore the relationship between URPED and CHRRPE.

## Case presentation

### Case 1

**Fig. 1:**
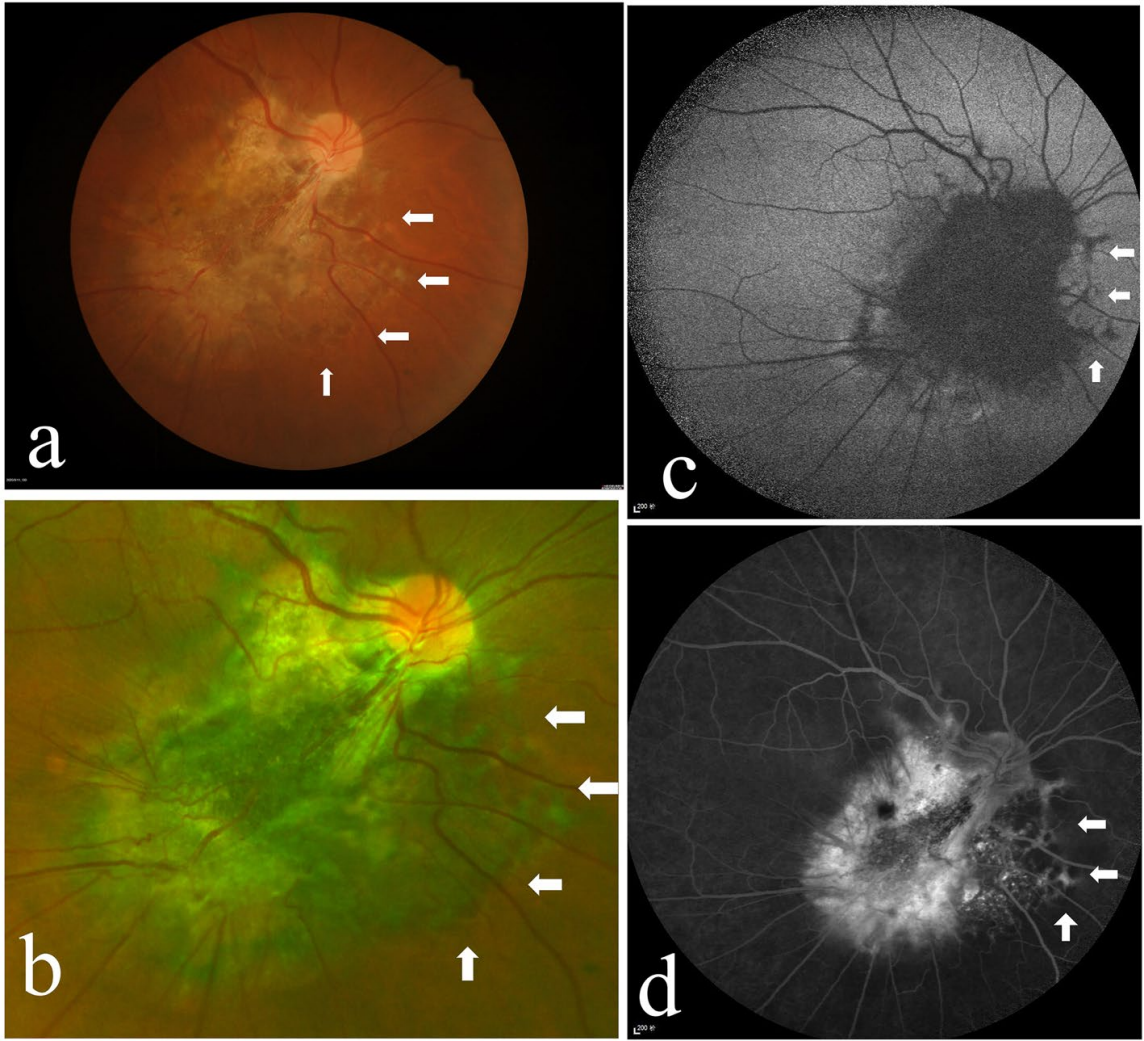
The right eye of the patient in Case 1. **a**, **b** The fundus photograph shows a large solitary yellowish-white lesion in the temporal and inferior parts of the optic disc. The lesion shows a scalloped reticular margin with hyperplastic retinal pigment epithelium and mild fibrosis (arrow). **c** Fundus autofluorescence (FAF) shows reticular hypoautofluorescence at the nasal margin of the lesion (arrow). **d** Fundus fluorescence angiography shows reticular hyperfluorescence, inverted compared with the results of FAF, at the nasal margin of the lesion (arrow)

**Fig. 2:**
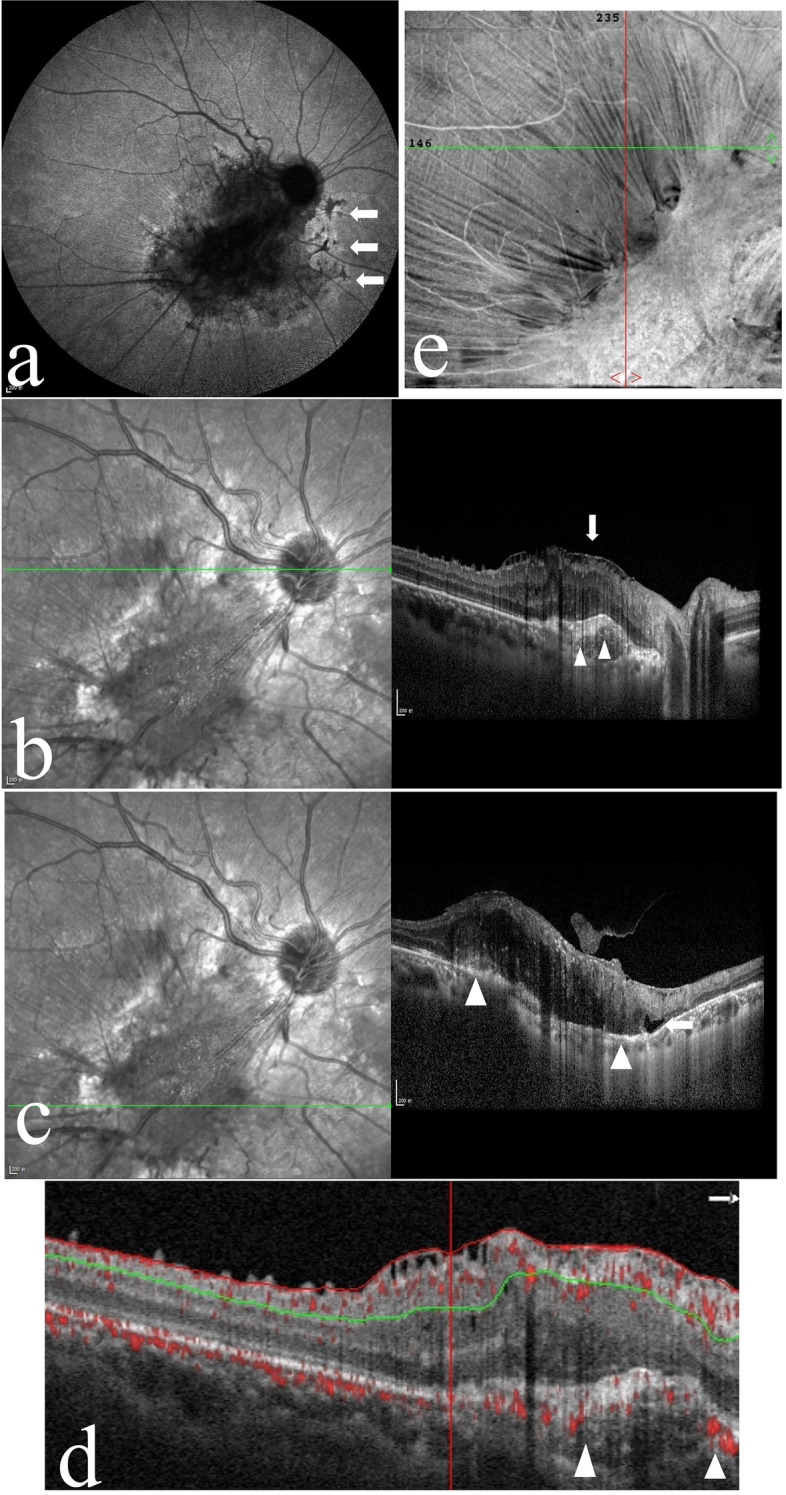
The right eye of patient in Case 1. **a** Indocyanine green angiography of the patient’s right eye shows reticular hypofluorescence at the nasal margin of the lesion, which was inverted compared with the results of FFA (arrow). **b**, **c** Optical coherence tomography (OCT) of the patient’s right eye. Figure b shows choroidal neovascularization (CNV, arrowhead) and epiretinal membrane (ERM, arrow) in the upper part of the lesion. Figure c reveals prominent disorganization of the retina involving the whole layer, ERM, RPE irregularities (arrowhead), and degenerative cystic changes (arrow). **d** OCT angiography shows abnormal blood flow signals at the CNV (arrowhead). **e** Enface OCT shows fibrous hyperplasia and retinal folds of the retina

A 51-year-old Chinese male complained of blurred vision and metamorphopsia in his right eye for the last 2 years. The patient’s visual acuity (VA) was 14/20 OD and 20/20 OS. He denied that he had experienced any ocular trauma. The anterior segment of both eyes was normal. Fundus examination of the right eye revealed a large solitary yellowish-white lesion on the posterior pole that connected with the optic disc and involved the macula. Fibroglial proliferation causing retinal vascular tortuosity and retinal folds were found in the lesion. Typical marginal reticular RPE fibrosis and hyperplasia scalloped at the inferior and nasal margins of the lesion (Fig. 1 a, b). FAF showed hypoautofluorescence in the centre of the lesion and reticular hypoautofluorescence at the nasal margin (Fig. 1 c). FFA depicted reticular hyperfluorescence at the nasal margin of the lesion, which was inverted relative to the results obtained with FAF (Fig. 1 d). In addition, indocyanine green angiography (ICGA) showed reticular hypofluorescence at the nasal margin of the lesions, which was inverted relative to the results obtained with FFA (Fig. 2 a). Optical coherence tomography (OCT) revealed CNV and ERM in the upper part of the lesion. The inferior part of the lesion showed prominent disorganization of the retina involving the whole layer and degenerative cystic changes. Hypertrophy and irregularity of the RPE were observed at the margin of the lesion (Fig. 2 b, c). An optical coherence tomography angiography (OCTA) scan revealed abnormal blood flow signals at the CNV in the upper part of the lesion and obvious fibrous hyperplasia and retinal folds of the retina on Enface OCT (Fig. 2 d, e).

**Fig. 3:**
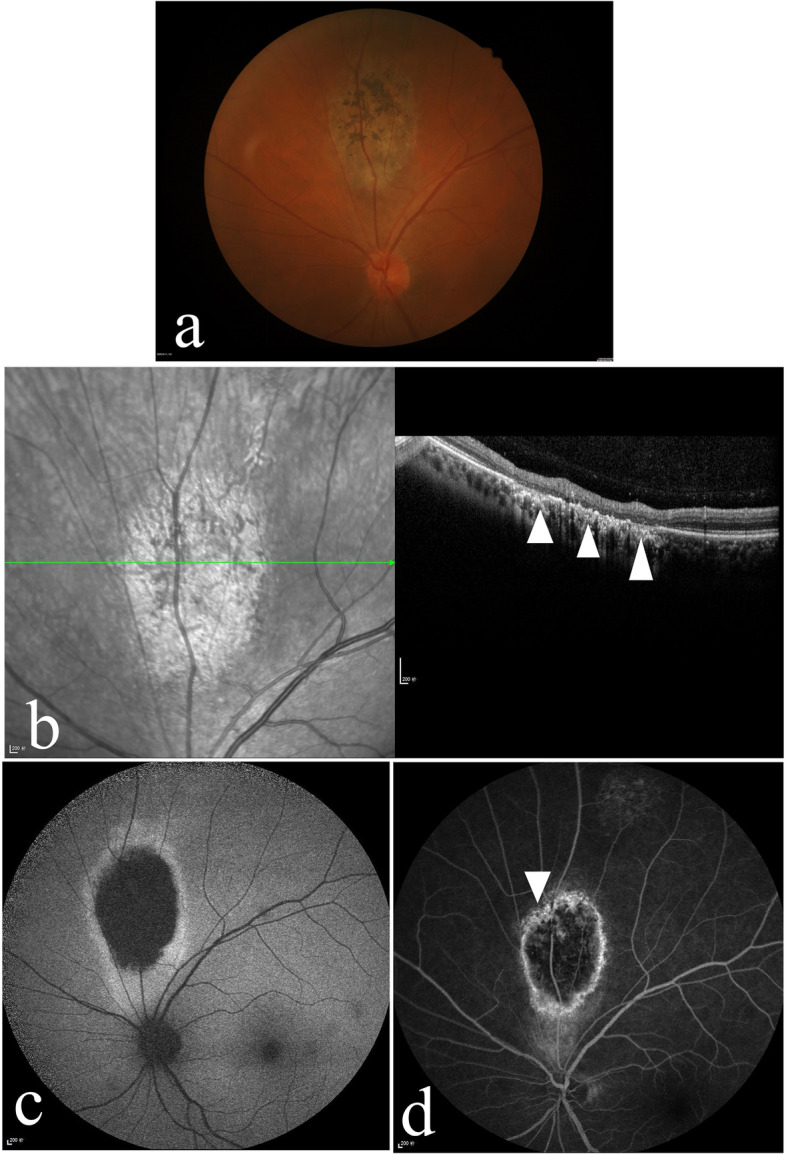
The left eye of the patient in Case 1. **a** The fundus photograph shows an oval-shaped lesion above the optic disc. **b** Optical coherence tomography depicted retinal pigment epithelium (RPE) layer atrophy with a small amount of RPE hyperplasia (arrowhead). **c** Fundus autofluorescence shows that the lesion was hypoautofluorescent and surrounded by hyperfluorescence. **d** Fundus fluorescence angiography shows hyperfluorescence in the margin of the lesion accompanied by a small amount of flaky hypofluorescence (arrowhead)

Additionally, an oval-shaped lesion at the RPE level was found above the optic disc of the patient’s left eye. The lesions were characterized by a defect of pigment with patchy hyperpigmentation (Fig. 3 a). OCT showed RPE layer atrophy with a small amount of RPE hyperplasia (Fig. 3 b). FAF depicted hypoautofluorescence in the centre of the lesion and hyperfluorescence surrounding the lesion (Fig. 3 c), and FFA revealed hypofluorescence in the centre of the lesion and hyperfluorescence in the margin, accompanied by a small amount of flaky hypofluorescence (Fig. 3 d).

### Case 2

**Fig. 4:**
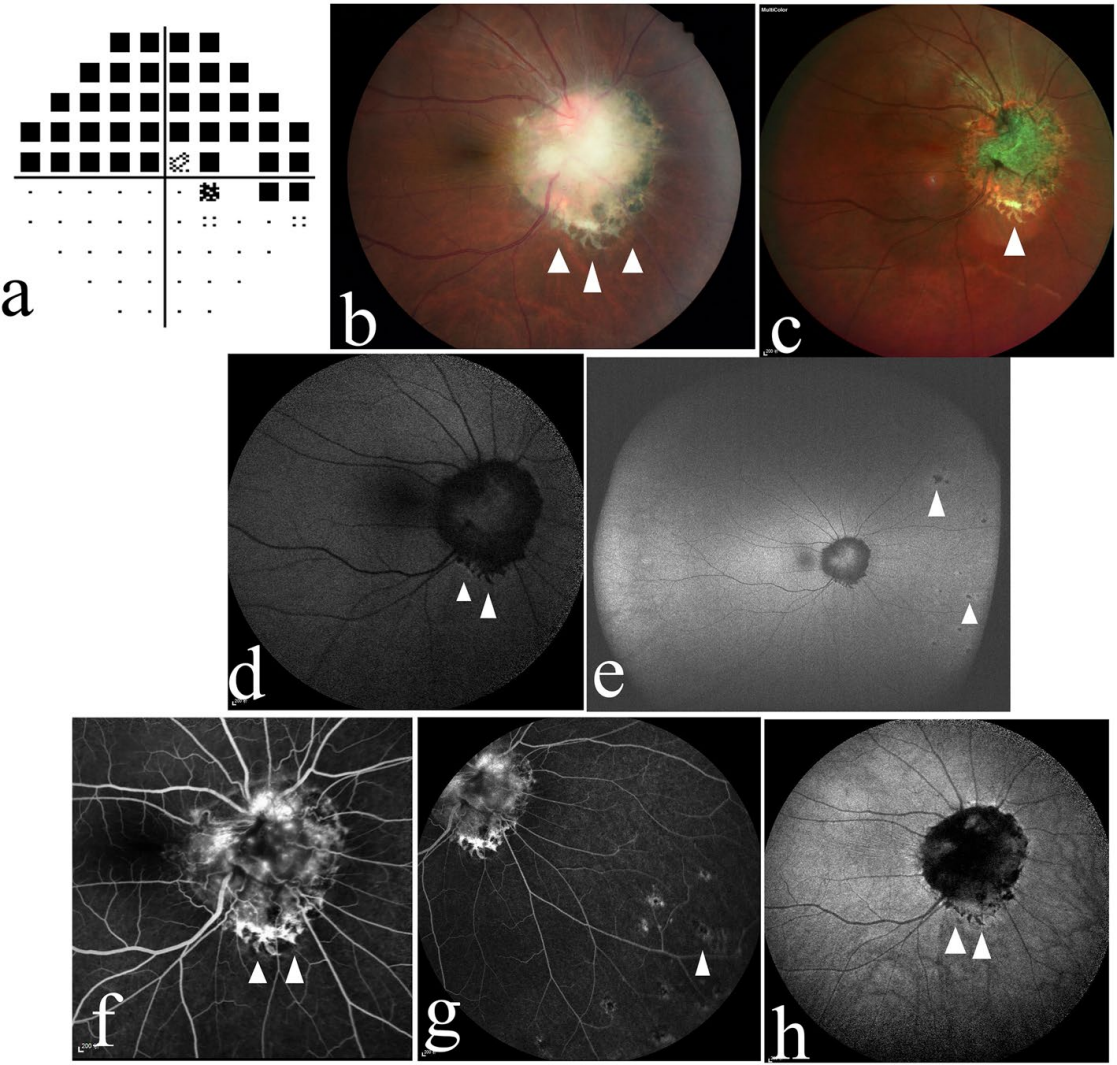
**a** The visual field examination of Case 2’s right eye demonstrates a defect in the upper part of the visual field. **b**, **c** The figures depict a solitary yellowish-white lesion under the optic disc that involved the optic disc, and the inferior margin of the lesion shows hyperplastic scalloped reticular margins of the retinal pigment epithelium and mild fibrosis (arrowhead). **d**, **e** Fundus autofluorescence of the patient’s right eye. Figure d shows reticular hypoautofluorescence at the inferior margin of the lesion (arrowhead), and small patches of hypoautofluorescence at the nasal side of the peripheral retina are observed in figure e (arrowhead). **f**, **g** Fundus fluorescence angiography shows reticular hyperfluorescence, inverted relative to the results of FAF, at the inferior margin of the lesion (fig. f arrowhead), and small patches of hyperfluorescence in the peripheral retina (fig. g arrowhead). **h** Indocyanine green angiography shows reticular hypofluorescence at the inferior margin of the lesion in the late stage, which was inverted compared with the results of FFA (arrowhead)

**Fig. 5:**
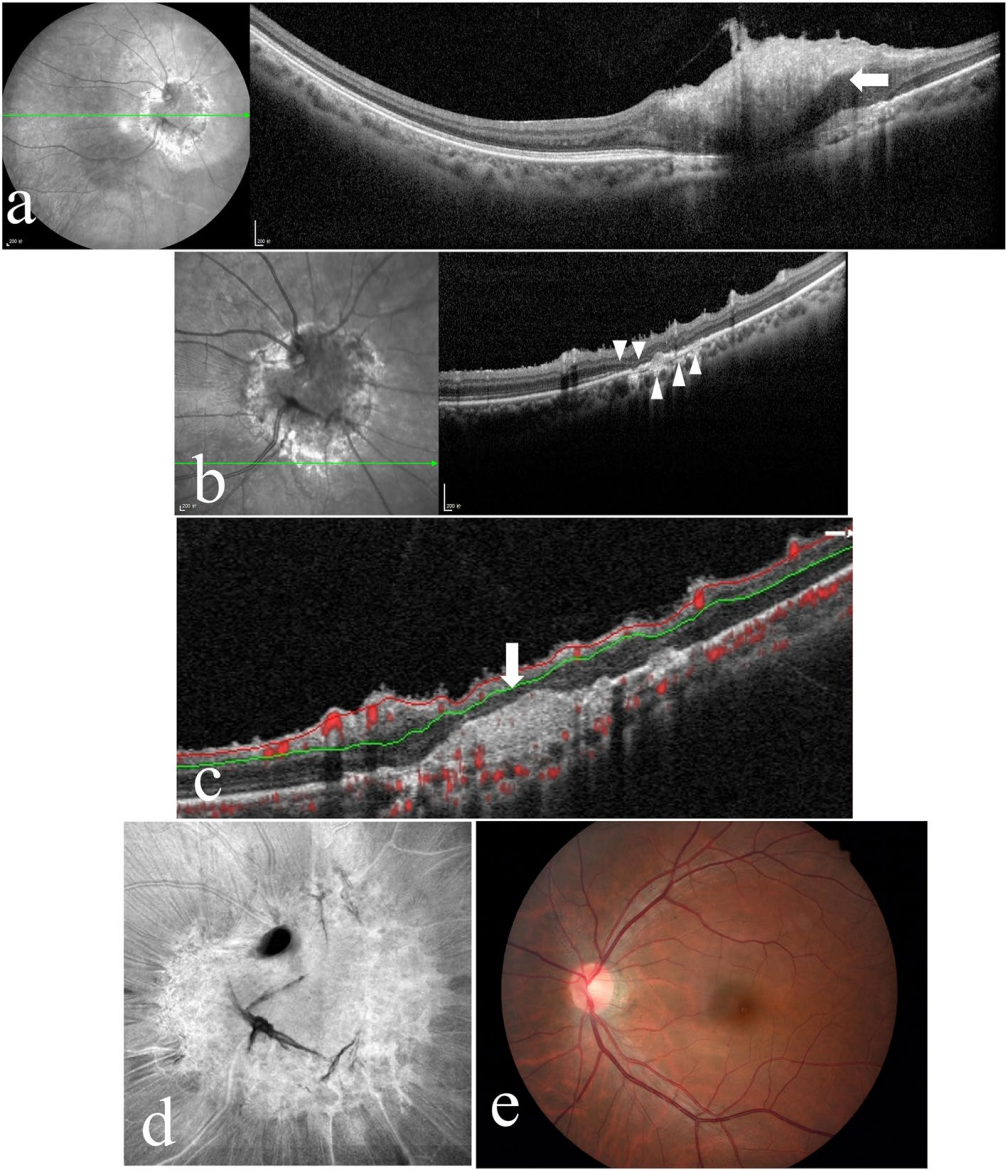
**a**, **b** Optical coherence tomography (OCT) of Case 2’s right eye. Figure a shows disorganization of the retina involving the whole layer (arrow). Figure b reveals ellipsoid zone disruption and RPE irregularities at the inferior margin of the lesion (arrowhead). **c** Optical coherence tomography angiography did not indicate abnormal blood flow signals in the subretinal hyperreflective materials (arrow). **d** Enface OCT of the patient’s right eye shows fibroglial proliferation and retinal folds. **e** The fundus photograph of the patient left eye is normal

A 39-year-old male Chinese patient was admitted to our hospital because he accidentally found visual field defects in his right eye. His VA was 20/25 OD and 20/20 OS, and his intraocular pressure (IOP) was 15 mmHg OD and 16 mmHg OS. He denied that he had experienced any ocular trauma, and the anterior segment of both eyes was normal. Visual field examination demonstrated a defect of the upper part of the visual field in the right eye (Fig. 4 a). Fundus examination depicted a solitary yellowish-white lesion under the optic disc of the right eye that involved the optic disc. Fibroglial proliferation, retinal vascular tortuosity, retinal folds, and a small amount of RPE cell proliferation and migration were observed. Typical scallop-like marginal reticular RPE fibrosis and hyperplasia were found at the inferior margin of the lesion (Fig. 4 b, c). In addition, there were small patches of abnormal RPE lesions in the nasal peripheral retina. FAF showed reticular hypoautofluorescence at the inferior margin of the lesion (Fig. 4 d). The peripheral retina of the nasal side showed small patches of hypoautofluorescence (Fig. 4 e). FFA revealed reticular hyperfluorescence at the inferior margin of the lesion, which was inverted relative to that observed with FAF, and small patches of hyperfluorescence in the peripheral retina (Fig. 4 f, g). ICGA revealed reticular hypofluorescence at the inferior margin of the lesion in the late stage, which was inverted relative to that observed with FFA (Fig. 4 h). OCT revealed prominent disorganization of the retina involving the whole layer. Subretinal hyperreflective materials were present in the lower lesions. An abnormal RPE layer was found at the inferior margin of the lesion (Fig. 5 a, b). OCTA did not indicate abnormal blood flow signals in the subretinal hyperreflective materials in the lower lesions, and significant fibroglial proliferation and retinal folds were shown by Enface OCT (Fig. 5 c, d). Fundus examinations of the patient’s left eye showed no abnormal changes (Fig. 5 e).

## Discussion and conclusions

URPED has nearly pathognomonic clinical features, namely, a scalloped reticular margin of hyperplastic RPE and mild fibrosis, with a unique inverted pattern on FAF and FA images [2]. In our study, there were corresponding manifestations at the nasal margin of the right eye lesion in Case 1 and at the inferior margin of the right eye lesion in Case 2 that could be used to diagnose both cases as URPED. Although there are obvious fundus features in URPED, it is important to address other similar clinical differential diagnoses. First, peripapillary acute zonal occult outer retinopathy is characterized by yellowish drusen-like material at the outer border of the lesion. The corresponding FAF imaging of the outer margin of the lesion shows intense hyperautofluorescence in a linear shape, which is significantly different from the reticular, fringe-like margin of URPED [7]. Second, the margins of congenital hypertrophy of the RPE are usually crisp, smooth, and regular, and the FAF is marked by dense hypoautofluorescence, frequently embedded with slightly hyperautofluorescent lacunae [8, 9], which can also be distinguished from URPED. Moreover, posttraumatic RPE hyperplasia can also have similar manifestations, but all patients in this study denied a history of ocular trauma [10]. Therefore, we think that the diagnosis of URPED in this study is correct.

Furthermore, an atrophic proliferative lesion at the RPE level was found above the optic disc of the left eye in Case 1. FAF showed that the centre of the lesion was hypoautofluorescent, which may be due to atrophy of the RPE. This reveals that RPE damage exists in both eyes in the patient in Case 1. The study of Benz et al. also found multifocal stellate lesions in both eyes, which further suggests that URPED may be a bilateral disease [11]. However, both the fellow eye in Benz et al.’s study and the left eye of Case 1 in our study lacked the characteristics of URPED. Therefore, we think that the viewpoint that URPED is a bilateral disease is not adequately supported and needs further research.

URPED was first proposed by Cohen et al. in 2009, and dysgenesis usually refers to organ defects or abnormal development [2]. However, other scholars suggest that the term “dysgenesis” may be incorrect or inaccurate due to the slow progress of the disease and that the disease may also be caused by inflammation [3, 4, 11]. Therefore, the aetiology of this condition remains elusive. The prognosis of this disease is also still unclear. Usually, patients maintain good VA for a long time, but when the lesion progresses into the macular region or is accompanied by complications such as CNV or ERM, visual impairment can occur [2–6]. In addition, the patient in Case 2 in our study was hospitalized due to a visual field defect. The patient’s VA was acceptable (20/25 OD, 20/20 OS), and defects in the upper half of the visual field were demonstrated by visual field examination, which has not been reported by any previous study. This suggests that URPED may cause visual field defects in patients with good VA when the optic disc is involved.

Recently, the relationship between URPED and CHRRPE has been controversial. Some studies postulated that URPED may be a forme fruste of CHRRPE [3], whereas others believe that there are obvious imaging differences between URPED and CHRRPE [2, 5, 6]. A recent comparative study found that peripapillary variants of CHRRPE are associated with a more severe degree of pigmentary changes and retinal disruption than those located in the macula [12]. CHRRPE in the macular area is less involved in the outer retina, such as the ellipsoid zone, and its imaging findings are easy to distinguish from those of URPED. However, peripapillary CHRRPE generally invades the outer retina and causes ellipsoid zone disruption and RPE irregularities, which are also manifestations of URPED [12]. Furthermore, disorganization of the retina involving the whole layer, which is common in peripapillary CHRRPE, was also seen with OCT in some previous URPED studies [3, 6]. Peripapillary CHRRPE and URPED have many other similar manifestations/complications, including retinal vascular tortuosity, ERM, retinal folds, and CNV [2, 12]. However, in other studies related to URPED, the URPED lesions were flat, and OCT showed a relatively normal structure of the neurosensory retina that was significantly different from that in peripapillary CHRRPE [11, 13, 14]. Therefore, we think that URPED may share some clinical features with peripapillary CHRRPE, and the relationship between URPED and CHRRPE needs further study.

In our study, although both cases had disorganization of the retina involving the whole layer, fibroglial proliferation, retinal vascular tortuosity, and retinal folds, the clinical manifestations were similar to those of CHRRPE. The two cases involved lesions that were flatter than those generally found in CHRRPE, and the neurosensory retina was relatively more preserved. The lesions showed a scalloped margin with fibrosis and focal hyperpigmentation of the RPE. The unique inverted pattern on FAF and FA images has been considered the distinguishing characteristic of URPED compared to CHRRPE and is shown on the nasal margin in Case 1 and on the inferior margin in Case 2 [14]. This has not been reported in related studies on CHRRPE, so the lesions in both cases are predominantly URPED rather than CHRRPE.

In conclusion, lesions in the affected eyes of the two URPED patients revealed many peripapillary CHRRPE imaging features. This suggests that URPED may share some clinical features with CHRRPE, and the relationship between peripapillary CHRRPE and URPED requires further research.

## Supplementary Information

**Additional file 1:** **Figure S1.** The right eye of the patient in case 1. (a) Fundus autofluorescence (FAF) shows reticular hypoautofluorescence at the nasal margin of the lesion (circle). (b) Figure b is a partial enlargement of the circle in the figure a. (c) Fundus fluorescence angiography (FFA) demonstrates well-defined reticular hyperfluorescent margins that are inverted compared with the results of FAF in the nasal part of the lesion surrounded by dark ovals (circle). (d) Figure d is a partial enlargement of the circle in figure c. (e) Indocyanine green angiography (ICGA) shows well-defined reticular hypofluorescent margins that inverted compared with the results of FFA in the nasal part of the lesion (circle). (f) Figure f is a partial enlargement of the circle in figure e.

**Additional file 2:** **Figure S2. **The right eye of the patient in Case 2. (a) Fundus autofluorescence (FAF) shows reticular hypoautofluorescence at the inferior margin of the lesion (circle). (b) Figure b is a partial enlargement of the circle in figure a. (c) Fundus fluorescence angiography (FFA) demonstrates well-defined reticular hyperfluorescent margins that are inverted compared with the results of FAF in the inferior part of the lesion (circle). (d) Figure d is a partial enlargement of the circle in figure c. (e) Indocyanine green angiography (ICGA) shows well-defined reticular hypofluorescent margins, inverted compared with the results of FFA, in the inferior part of the lesion (circle). (f) Figure f is a partial enlargement of the circle in figure e.

## Data Availability

The datasets used and/or analyzed during the current study are available from the corresponding author on reasonable request.

## Abbreviations

URPED: Unilateral retinal pigment epithelium dysgenesis
RPE: Retinal pigment epithelium
FAF: Fundus autofluorescence
FFA: Fundus fluorescence angiography
CNV: Choroidal neovascularization
CHRRPE: Combined hamartoma of the retina and the RPE
ICGA: Indocyanine green angiography
OCT: Optical coherence tomography
OCTA: Optical coherence tomography angiography
ERM: Epiretinal membrane
